# Haplotype Analysis Improved Evidence for Candidate Genes for Intramuscular Fat Percentage from a Genome Wide Association Study of Cattle

**DOI:** 10.1371/journal.pone.0029601

**Published:** 2011-12-28

**Authors:** William Barendse

**Affiliations:** 1 Cooperative Research Centre for Beef Genetic Technologies, Commonwealth Scientific and Industrial Research Organization, St. Lucia, Queensland, Australia; 2 School of Veterinary Science, University of Queensland, Gatton, Queensland, Australia; University of Munich, Germany

## Abstract

In genome wide association studies (GWAS), haplotype analyses of SNP data are neglected in favour of single point analysis of associations. In a recent GWAS, we found that none of the known candidate genes for intramuscular fat (IMF) had been identified. In this study, data from the GWAS for these candidate genes were re-analysed as haplotypes. First, we confirmed that the methodology would find evidence for association between haplotypes in candidate genes of the calpain-calpastatin complex and *musculus longissimus lumborum* peak force (LLPF), because these genes had been confirmed through single point analysis in the GWAS. Then, for intramuscular fat percent (IMF), we found significant partial haplotype substitution effects for the genes *ADIPOQ* and *CXCR4*, as well as suggestive associations to the genes *CEBPA*, *FASN*, and *CAPN1*. Haplotypes for these genes explained 80% more of the phenotypic variance compared to the best single SNP. For some genes the analyses suggested that there was more than one causative mutation in some genes, or confirmed that some causative mutations are limited to particular subgroups of a species. Fitting the SNPs and their interactions simultaneously explained a similar amount of the phenotypic variance compared to haplotype analyses. Haplotype analysis is a neglected part of the suite of tools used to analyse GWAS data, would be a useful method to extract more information from these data sets, and may contribute to reducing the missing heritability problem.

## Introduction

Genome wide association studies (GWAS) almost invariably use single point analysis [1], [2], [3] despite the potential for increased levels of information that can be achieved by the analysis of haplotypes [4], [5], [6]. Single point analyses are logistically and statistically simple, because 1) the single nucleotide polymorphisms (SNPs) can be analysed one at a time and genomic information can be supplied later to order the p-values along a chromosome, and 2) each SNP is tested once and the significance threshold can be easily adjusted for the number of independent tests performed, a threshold that is partly determined by the degree of linkage disequilibrium between SNPs along the chromosome and the size of the genome of the species.

There are several difficulties with haplotype analyses that have resulted in their rare use in GWAS. Firstly, there is no strong consensus on how haplotypes should be analysed, with several methods resulting in the double counting of individuals because they have more than one haplotype [4], [5], [6], [7], [8], [9], [10], [11], [12], [13], [14], [15], [16]. Secondly, there is the question of how many SNPs or other polymorphisms should be in a haplotype, while determining which is the most significant haplotype involves an exploratory analysis, both processes that result in a large number of additional tests being performed [17]. In the context of a GWAS, there is no clear consensus about whether these additional tests would need to be accounted for in setting the threshold for significance [2], [18], [19], [20], [21], [22], [23], [24], [25], [26]. Thirdly, simulation results have shown that if haplotypes contain causative SNPs then the advantage of haplotype analysis in general over single point analysis may be slight [27], [28], although most panels of SNPs for GWAS do not contain large numbers of causative mutations. These factors have led to the growth of strategies for imputation of genotypes, which is a complementary aspect of multimarker analyses compared to haplotype analysis [29]. Effectively, the array of SNPs becomes substantially larger. Ultimately the genome of an individual could be imputed based on a SNP array genotype if many individuals in the population have been genome sequenced [30], [31], [32], and therefore, imputed causative mutations could be tested rather than mere DNA markers.

There are nevertheless good reasons to perform haplotype analyses to test for associations. Firstly, most risk loci for complex or quantitative traits appear to have small to very small effects [3], [33], [34] but there is also evidence that some QTL may be grouped into haplotypes that have larger aggregated effects [35]. On the contrary, there is also evidence of rare genetic effects of large effect that are clustered together on haplotypes and these generate synthetic associations that are interpreted instead as common genetic variation of small effect [36]. In addition, one could reconcile the oligogenic effects detected by family linkage analysis [37] with the polygenic effects detected by GWAS by postulating that the polygenes have been aggregated together to make a haplotype that is oligogenic in size of effect and which is inherited as a block within families, because recombination fails to break up the haplotype within the time scale of most human linkage studies. The analysis of haplotypes would help to distinguish between these alternative scenarios. Secondly, few species will have the necessary resources in the short term to integrate high density arrays with genome sequencing so that full genome sequences can be imputed for large numbers of individuals. This may only become available for humans and a small number of agriculturally important species such as cattle.

Another feature of GWAS is that they are considered to be agnostic to the genetic basis of a trait, so that one does not focus only on the genes likely to affect the phenotype but on all possible parts of the genome. The surprising finding from many GWAS is the lack of association between many good candidate genes and their cognate traits, and the discovery of a wide range of genomic regions, some containing no genes, that have reproducible and small effects on traits. Indeed, this has prompted some suggestions that a two tier system should be introduced, one for variants in candidate genes and one for random variants in or near other genes [38], with different significance thresholds or *a priori* Bayesian weighting for the two types of SNPs. One could imagine other tiers, dependent upon whether, for example, the variation deleted genes, altered splicing, transcription, or amino acid substitution, or resulted in purely neutral DNA markers [39], [40]. All of these point to an interesting feature of GWAS, that so far they have identified little of the genetic variance for most traits, accounting for amounts of variance and identities of associations that are inconsistent with previous research [41], [42], [43].

In a recent GWAS of intramuscular fat percentage (IMF) [44] using the Illumina Bovine SNP50 array, we found that none of the previously identified candidate genes for this trait showed an association to the trait despite the fact that several SNPs were associated with IMF above the significance threshold and were confirmed in a separate sample. Indeed, none of the confirmed SNPs was close to a well studied, candidate or positional candidate gene for fatness, nor were any of these confirmed genes identified as top candidates in gene expression studies [45]. On the other hand, for a second trait, *musculus longissimus lumborum* peak force (LLPF), evidence for associations to the candidate genes calpain 1 (*CAPN1*) and calpastatin (*CAST*) in the calpain-calpastatin pathway [46], [47], [48], [49] was found in the discovery sample of the GWAS as well as in the confirmation sample. The lack of association of candidate genes to IMF could be due to several factors. First, there could have been differences in the size of effect of the SNPs, so that those for LLPF were detectable but those for IMF were not. Second, the QTL may not have been segregating at a sufficiently high frequency to be detected. Third, there might have been a difference in the density of coverage of SNPs on the array for the candidate genes for IMF and LLPF, or differences in the degree of LD across the region. In this regard, one should note that the CAPN1_1 SNP in the Illumina Bovine SNP50 array is the only SNP in a candidate gene for these two traits which has any claim to being a causal mutation, and along with CAPN1_2, were the only SNPs in the SNP array in candidate genes that were known to be associated to either LLPF or IMF in previous studies. None of the candidate genes for IMF were represented by the SNPs that were previously found significantly associated with IMF. Analysis of haplotypes in candidate genes is therefore a plausible approach for further investigation of the lack of association of candidate genes to IMF in this GWAS.

In this study, 3-SNP haplotypes around candidate genes for IMF and LLPF were analysed using data from a recent GWAS study to determine whether haplotype analysis provided more evidence for associations than single SNP analysis. SNPs in genes of the calpain-calpastatin pathway were used to test the methods, because single SNP analyses had been successful in identifying associations. Then SNPs in candidate genes for IMF were examined, because none of the candidate genes that had previously been identified for this trait had been found associated to the trait in the GWAS. We found evidence for two of the genes, and suggestive evidence for three other genes. 3-SNP haplotype analyses explained more of the phenotypic variance than analysing the 3 SNP simultaneously, although models that included the interactions between SNPs accounted for essentially the same amount of variance as haplotypes. While haplotype analysis did provide additional evidence for these candidate genes, other factors, such as presence of informative SNP at the candidate genes, SNP density, and genetic background of the samples are alternative explanations for the lack of association of some of the candidate genes to IMF in the GWAS.

## Results

First, we characterised the trait distribution for IMF and LLPF in the animals of the sample. There were significant differences between breeds in the level of IMF and LLPF in the animals used in this study, and a substantial part of the heritability was partitioned between breeds. As expected, the taurine breed samples showed a higher percentage of IMF on average than composite or indicine breed samples, and although there was overlap between individuals of different breeds, there were distinct overall differences between the breed samples in distribution of IMF (Table S1), with F_11,851_ = 10.98, *P* = 0. The taurine breed samples also required lower amounts of peak force to shear the meat samples, leading to more tender meat, and there were distinct overall differences in distribution of LLPF between the breed samples (Table S2), with F_11,847_ = 4.48, *P* = 1.34e-06. When adjusted for breed and ancestry, the narrow sense heritability of IMF in this sample was *h* ^2^ = 0.47 (s.e. 0.13) and of LLPF was *h* ^2^ = 0.12 (s.e. 0.11). However, as breed encapsulates genetic differences, when ancestry but not breed was fitted in the model the narrow sense heritability of IMF was *h* ^2^ = 0.75 (s.e. 0.13) and of LLPF was *h* ^2^ = 0.32 (s.e. 0.12). This shows substantial additive genetic variance between breeds for these two traits.

The SNPs near the candidate genes showed strong differences in allele and haplotype frequencies between breeds (Table S3). One SNP by breed combination, ARS-BFGL-NGS-101028 in the SGT breed, showed a departure from HWE (G_adj_ = 6.89, df = 1, P = 0.032), or 1 out of the 66 by 7 breed tests or 0.2%. This was low compared to the 1.75% of the breed by SNP tests in the entire GWAS that had *P*<0.05. Only one SNP, ARS-BFGL-BAC-21527, did not show a significant allele frequency difference between breeds, and the minor allele frequency (MAF) ranged from 0.00 to 0.01. All other SNPs (Table S3) showed significant allele frequency differences between breeds with *P*<0.001. In the entire GWAS dataset only 4823 of the SNPs had reasonably similar allele frequencies, with *P*>0.001, all other SNPs showed highly divergent allele frequencies. Consistent with this, the distribution of F_ST_ for the SNPs in the 7 pure breeds showed mean F_ST_ = 0.13 (s.d. = 0.07, n = 50,625) with the top 2.5% corresponding to a threshold of F_ST_ = 0.292 and a bottom 2.5% to a threshold of F_ST_ = 0.018. The SNP ARS-BFGL-NGS-4939 (*DGAT1*) and Hapmap49048-BTA-119203 (*TCAP*) exceeded the top threshold with F_ST_ = 0.325 and F_ST_ = 0.295 respectively. Overall, the SNPs near the candidate genes showed mean F_ST_ = 0.14 (s.d. = 0.08, n = 66), similar to the full distribution of SNPs in the GWAS. The haplotype frequency differences were also consistent with the scale of the allele frequency differences between breeds. All gene by breed haplotype frequency tests (Table S4) showed significant differences with *P*<<0.0001. This applied whether all breeds were compared or only the taurine breeds.

**Figure 1:**
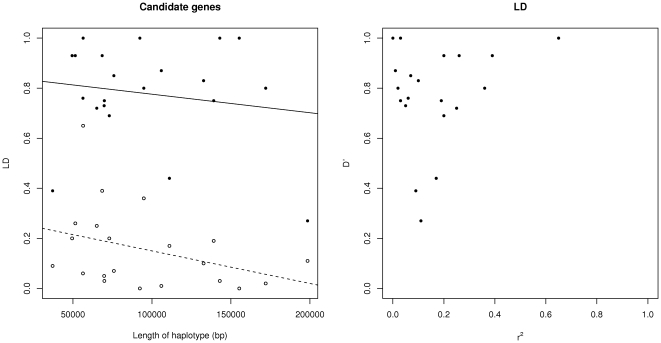
Comparison of linkage disequilibrium (LD) measures for the genes in the study. **A. D′ and** ***r*** **^2^ plotted against distance between SNPs.** D′ values are filled black circles, *r* ^2^ values are open black circles. Least squares fitted regression lines of LD on length of haplotype (D′ solid line, *r* ^2^ dashed line) are not statistically significant and the slopes are *b*<−1×10^−5^. This is evidence that the length differences between haplotypes are not important in accounting for LD between SNPs in this sample of genes. Values are means of LD estimates for each breed, not calculated from a sample of mixed breed individuals. **B. Plot of D′ against** ***r*** **^2^ for the genes in this study.** Most of the comparisons between pairs of SNPs show high D′and low *r* ^2^ values, a typical result for cattle at this distance between SNPs. High D′values can indicate a reduced number of haplotypes or classes of haplotypes that are missing. *r* ^2^ values are useful in describing how well the genotypes at one SNP predict the genotypes at the other SNP.

To determine whether the haplotypes had different levels of LD related to their length, two measures of LD were calculated between the SNPs of each gene for each breed. For the haplotype lengths in this sample, from 37,111 bp to 198,517 bp (Figure 1A), the degree of LD, either measured as D′ or as *r* ^2^, was not significantly related to haplotype length. Although the slope of the least squares regression was negative, as expected, in this range of haplotype lengths and for these genes, the slope was not significantly different to zero. The most obvious pattern for pairs of these SNPs was a high D′ value and a low *r* ^2^ value within each breed (Figure 1B). We used *r*, the square root of *r* ^2^, for the two SNPs at either end of the haplotype to determine whether the same orientation of haplotypes occurs for most breeds, as a measure of haplotype structure. Of the 7 purebreeds, 19 genes had sufficient information for the two SNPs at the end of the haplotype, and 11 of those had 80% of the breeds with the same polarity (i.e., plus or minus) for their *r* values. Some long haplotypes had all breeds with the same polarity of *r* and some short haplotypes had half the breeds of opposite polarity (Table S5). Again, haplotype length was not strongly related to haplotype structure for this sample of haplotypes and genes. Finally, when comparing the length of haplotypes that were significant associated to IMF to those that were not significantly associated to IMF, we found that although the mean difference of significant haplotypes was shorter by 24.4 kb on average than non-significant haplotypes, this difference was not statistically significant (t = 1.70, n.s). The average differences in D′ (−0.037, t = 0.06) and *r* ^2^ (−0.071, t = 1.15) for loci associated to IMF minus those not associated to IMF were also not statistically significant.

Before analysing the relationships of IMF to haplotypes, the relationships between LLPF and haplotypes at the calpain-calpastatin genes were analysed as a check of the methods. SNPs in and around *CAPN1* and *CAST* have repeatedly been found associated to different objective measurements of meat tenderness including the LLPF measurement in this study (see above). In the original GWAS, the SNP CAPN1_1, a non-synonymous substitution in *CAPN1*, was significantly associated to LLPF (Table 1). Although the SNP CAPN1_2, an intronic, non-functional SNP segregating with a QTL in indicine cattle, showed suggestive evidence in this sample, it did not pass the *P* = 0.001 threshold. In addition, none of the SNPs in or near to *CAST* or calpain 3 *(CAPN3)* showed evidence of association to LLPF even at a more relaxed threshold of *P* = 0.05. The SNP *CAST*:c.2832A>G, which is not part of the Bovine SNP50 array, had shown a significant (*P*<0.001) association to LLPF in the Beef CRC cattle, so the QTL is known to be segregating in this sample. SNPs for the gene *CAPN3* had previously shown a weaker effect on LLPF, but none of those SNPs was part of the Bovine SNP50 array, and the association appeared to be found only in indicine and indicine derived cattle. The calpain-calpastatin genes therefore act as a series of graded difficulty, *CAPN1* was represented by one of the putative causative mutations, *CAST* had none of the putative mutations but the QTL is known to be segregating in the sample, and the weaker QTL at *CAPN3* is thought to be restricted to indicine breeds.

**Table 1:** SNPs of the calpain-calpastatin gene haplotypes associated as single point associations to LLPF in the GWAS.

SNP	A	B*	Bta	Position (bp)	*R* ^2^ (%)	*b* † (kg)	s.e.‡	*P*
*CAST*								
ARS-BFGL-NGS-43901	A	C	7	97492911	0.0	−0.026	0.040	0.5171
ARS-USMARC-670	A	G	7	97524770	0.1	0.061	0.037	0.0952
ARS-USMARC-116	A	G	7	97561407	0.1	0.065	0.040	0.1059
*CAPN3*								
ARS-BFGL-NGS-13350	A	G	10	37625930	0.1	0.026	0.038	0.4841
Hapmap47063-BTA-62293	A	G	10	37647411	0.0	0.032	0.038	0.3931
ARS-BFGL-BAC-12264	A	G	10	37675399	0.0	0.007	0.056	0.9204
*CAPN1*								
ARS-BFGL-NGS-21416	A	G	29	45202710	0.1	0.046	0.046	0.3176
CAPN1_1	C	G	29	45221190	1.1	0.179	0.040	8.8e-06
CAPN1_2	A	G	29	45239821	0.9	−0.138	0.043	0.0012

*Regressions were performed on number of copies of the B allele.

† *b* regression coefficient of LLPF regressed on number of B allele copies.

‡ s.e. standard error of *b*.

In this graded series of calpain-calpastatin genes, evidence for associations to LLPF were found for all three genes (Table 2) even though associations could only be demonstrated for one of the three genes using the single point analysis (Table 1). The standard analysis in this study is for all haplotypes with minor haplotype frequency (MHF)≥0.05 to be analysed simultaneously. For *CAPN1*, where the middle SNP of the haplotype is one of the putative causal mutations, the haplotype h112 shows a significant (*P*<0.001) association to LLPF. When this haplotype was analysed by itself the amount of variance explained more than doubled, despite the presence of a putative causative mutation. Haplotype h112 is the only haplotype that contains the C allele of CAPN1_1. There are 4 haplotypes that contain the G allele of CAPN1_1, and when these are analysed in the absence of h112, in effect partitioning the G allele into subgroups, the haplotype h121, which had not been significant in the analysis containing h112, became significant at *P* = 0.001. This suggested that some of the variability associated with h112 was represented by h121 in the absence of h112, but that not all of the subgroups of allele G were equally significantly associated to LLPF. For *CAST*, the standard analysis of haplotypes showed some suggestive evidence for an association of this gene to LLPF, where the haplotype h222 showed an association with *P* = 0.0042. The results for *CAST* are not as clear as for *CAPN1*, partly because none of the SNP in the haplotype was either one of the causal alleles or in very strong LD to the causal alleles. Nevertheless, the haplotype analysis was able to extract more information out of the data than using the SNP by themselves, all of which had shown *P*>0.05 when tested against LLPF. Analysing the haplotypes together was also more powerful than analysing haplotypes one at a time, haplotype h222 showed weaker results when analysed by itself. For *CAPN3*, the standard analysis, namely, all haplotypes with MHF>0.05 analysed simultaneously, showed no effect of the haplotypes on LLPF. This sample contained only 78 BRM animals, so it is possible that the effects of the QTL are not visible because most of the breeds were not segregating it. Using the most common haplotype, h211, the haplotype effect was estimated by breed. The effect on LLPF in the BRM breed was significant (*P* = 1.8e-05), consistent with previous results [50], but in this case, the use of 3-SNP haplotypes revealed stronger evidence than was previously presented using 2-SNP haplotypes. This effect could still be seen when all haplotypes with MHF>0.05 were analysed simultaneously partitioned by breed, where the effect was still significant (*P* = 2.1e-05).

**Table 2:** Calpain-calpastatin gene haplotypes associated to LLPF.

Haplotype	*R^2^* (%)	*b* * (kg)	s.e.†	*P*
*CAPN1*				
excluding MHF<0.05				
h222‡	3.4	−0.238	0.125	0.0584
h221		−0.302	0.124	0.0151
h122		−0.241	0.113	0.0323
h121		−0.154	0.110	0.1596
h112		−0.418	0.114	0.0002
excluding h112	1.5			
h222		0.136	0.074	0.0682
h221		0.067	0.075	0.3753
h122		0.121	0.056	0.0297
h121		0.197	0.056	0.0005
only h112				
h112	2.2	−0.206	0.047	1.15e-05
*CAST*				
excluding MHF<0.05				
h222	1.6	0.397	0.138	0.0042
h212		0.360	0.149	0.0161
h211		0.221	0.127	0.0819
h122		0.211	0.132	0.1122
h121		0.338	0.147	0.0216
only h222				
h222	0.9	0.162	0.075	0.0319
*CAPN3*				
excluding MHF<0.05				
h222	0.4	−0.186	0.202	0.3561
h221		−0.151	0.191	0.4292
h211		−0.193	0.191	0.3135
h122		−0.243	0.202	0.2298
h121		−0.208	0.190	0.2751
h211 analysed by breed				
ANG	3.1	−0.005	0.079	0.9476
HFD		−0.088	0.107	0.4151
MGY		−0.184	0.154	0.2327
SHN		−0.084	0.135	0.5362
BEL		0.052	0.095	0.5850
SGT		−0.049	0.122	0.6861
BRM		2.029	0.471	1.8e-05

*b regression of LLPF on number of copies of the haplotype.

† s.e. standard error of b.

‡ h111 is the haplotype of all the A alleles (AAA) while h222 is the haplotype of all the B alleles (BBB) see Table 1 for the code of A and B alleles.

Having found that 3-SNP haplotype analyses of LLPF helped to increase the amount of variance explained, we used this approach to examine whether there was an increase in evidence for candidate genes for IMF. First, the SNP associations to IMF from the original GWAS study were inspected. None of the SNPs from the candidate or positional candidate genes that had previously been studied for IMF was significantly associated (*P*<0.001) to IMF (Table 3 & S6). Given the threshold in the GWAS, only one of the genes in this study, *CXCR4*, had been examined further in the confirmation sample of the original GWAS, with successful confirmation. Nevertheless, *CXCR4* is merely the closest gene to the SNPs showing the significant associations, which are not in the *CXCR4* gene itself, and when the region was first identified the assembly at the time did not identify a gene near to the SNPs. This region was chosen as an example of a region without candidate genes (see Discussion) for confirming the methods, but the new assembly placed the SNPs close to a plausible candidate gene. To simplify description, the SNPs near *CXCR4* are identified as the *CXCR4* SNPs. Several of the SNPs in this study had p-values in the range 0.05>*P*≥0.001 when tested in the single point analysis in the GWAS. The SNPs with the best suggestive evidence were located near *CXCR4*, where one of the SNP, Hapmap55796-rs29011172, was associated with *P* = 0.0016 to IMF and a second SNP was associated 0.02>*P*>0.01 to IMF in the GWAS. For the gene *CEBPA*, the SNP ARS-BFGL-NGS-21339, was associated to IMF with *P* = 0.009 in the GWAS. For the gene *ADIPOQ*, two of the SNPs that formed the 3-locus haplotype in this study showed associations with 0.02>*P*>0.01 to IMF in the GWAS. One of the *CAPN1* SNP, CAPN1_1, showed suggestive evidence with *P* = 0.0348. All other SNP in the 3-SNP haplotype of the genes in this study showed associations with *P*>0.05 in the GWAS.

**Table 3:** Single point SNP associations of candidate genes for IMF in the GWAS.

SNP	A	B	Bta	Position (bp)	*R^2^* (%)	*b* (%)	s.e.	*P*
***ADIPOQ***								
ARS-BFGL-NGS-26946	A	G	1	82201457	0.0	0.049	0.103	0.6316
Hapmap43250-BTA-37524	A	G	1	82245379	1.4	−0.956	0.378	0.0117
BTB-00035080	A	G	1	82271202	1.4	−0.642	0.274	0.0191
***CXCR4***								
ARS-BFGL-NGS-117383	A	G	2	63905821	0.0	0.331	0.413	0.4239
Hapmap55796-rs29011172	A	T	2	63947669	1.1	−0.427	0.135	0.0016
ARS-BFGL-NGS-119079	A	G	2	63998173	0.9	0.297	0.116	0.0107
***CEBPA***								
ARS-BFGL-NGS-105692	A	G	18	43119331	0.0	−0.076	0.176	0.6715
ARS-BFGL-NGS-21339	A	G	18	43150185	1.4	0.265	0.101	0.0092
BTA-43268-no-rs	A	G	18	43170819	0.1	−0.188	0.123	0.1273
***CAPN1***								
ARS-BFGL-NGS-21416	A	G	29	45202710	0.1	−0.019	0.104	0.8625
CAPN1_1	C	G	29	45221190	0.2	−0.192	0.091	0.0348
CAPN1_2	A	G	29	45239821	0.0	−0.008	0.099	0.9204

This list consists of all the genes with at least 1 SNP with *P*<0.05 to IMF, the full list is in the supplementary online material.

Note that for the gene *CXCR4*, this gene is the closest gene to the significant SNPs, but these are not located within the gene itself.

**Figure 2:**
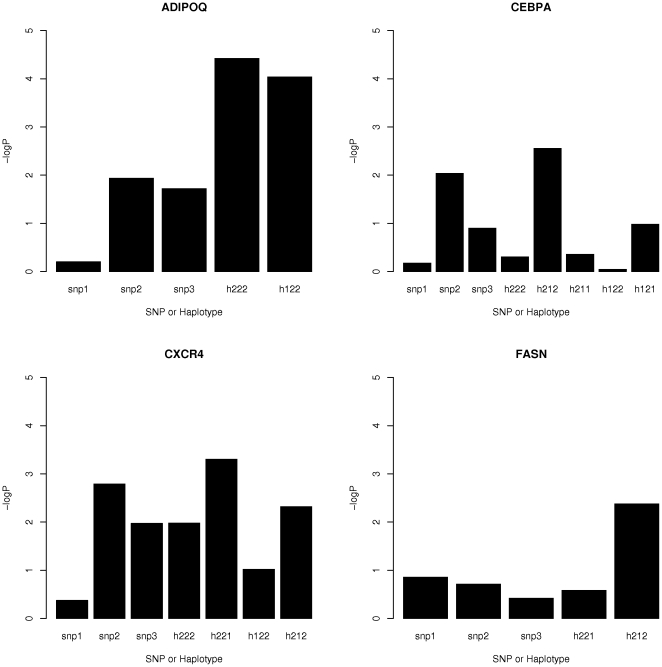
Plot of –logP values for SNPs compared to haplotypes for candidate genes for IMF. The SNPs are numbered 1, 2, and 3 in order along the chromosome and in the haplotypes, 1 = A and 2 = B alleles at each SNP. Haplotypes were fitted simultaneously. Note that for the gene *CXCR4*, this gene is the closest gene to the significant SNPs, but these are not located within the gene itself.

There was more evidence for association in the analysis of haplotypes for some of the candidate genes for IMF (Table 4 & S7). For example, for *ADIPOQ* and *CXCR4*, the common haplotypes were significantly (*P*<0.001) associated to IMF (Figure 2). There was a slight improvement in evidence for *CXCR4* and a major improvement for *ADIPOQ*. For *CEBPA* and *FASN* there was also an improvement in the amount of support (*P*<0.01) for the association. In the case of *FASN*, none of the SNPs in the haplotype showed associations (*P*<0.05) to IMF in single SNP analyses in the original analyses (Table S6), but imputation of missing data for 17 individuals for SNP ARS-BFGL-NGS-20701 resulted in an association to IMF with *b* = −0.313, s.e. = 0.111, *P* = 0.0049, which accounted for 0.8% of the phenotypic variance. One haplotype for *CAPN1*, h122, did show an association (*P* = 0.01) to IMF, when a subset of the haplotypes minus h112 (see section on LLPF haplotypes) was analysed. This haplotype decreased LLPF and increased IMF at the same time. For the other candidate genes for IMF, none showed individual SNP or 3-SNP haplotypes associated to IMF even at a threshold of *P* = 0.05.

**Table 4:** Haplotype associations of candidate genes for IMF in the GWAS.

Haplotype	*R* ^2^ (%)	*b* (%)	s.e.	*P*
***ADIPOQ***				
h222	2.4	−0.985	0.238	3.8e-05
h122		−0.879	0.224	9.2e-05
***CXCR4***				
h222	1.7	−0.307	0.120	0.0105
h221		−0.518	0.148	5.0e-04
h122		−0.485	0.291	0.0960
*or*				
h212	1.0	0.337	0.119	0.0048
***CEBPA***				
h221	1.9	0.192	0.284	0.4997
h212		−0.292	0.097	0.0028
h211		−0.126	0.163	0.4413
h122		0.026	0.212	0.9007
h121		−0.374	0.231	0.1054
*or*				
h212	1.5	−0.236	0.078	0.0027
***FASN***				
h222	1.8	0.302	0.224	0.1781
h221		0.224	0.205	0.2737
h212		0.051	0.205	0.8018
h121		0.338	0.264	0.2004
h112		0.519	0.229	0.0238
*or*				
h221	1.3	−0.102	0.091	0.2623
h212		−0.287	0.100	0.0042
*or*				
h212	1.2	−0.239	0.090	0.0081
***CAPN1***				
h222	1.0	0.168	0.232	0.4674
h221		0.156	0.230	0.4985
h122		−0.102	0.208	0.6230
h121		0.080	0.203	0.6936
h112		0.186	0.210	0.3762
*or*				
h222	1.0	0.003	0.135	0.9847
h221		−0.007	0.138	0.9564
h122		−0.263	0.102	0.0100
h121		−0.074	0.102	0.4684

This list consists of all the genes with at least 1 haplotype with *P*<0.05 to IMF, the full list is in the supplementary online material.

Note that for the gene *CXCR4*, this gene is the closest gene to the significant SNPs, but these are not located within the gene itself.

It is possible that these candidate genes were associated to IMF due to variation in genes adjacent to the candidate genes rather than variation at the candidate genes. To test this possibility, 3-SNP haplotypes flanking the SNPs of *ADIPOQ*, *CAPN1*, *CEBPA*, *CXCR4*, and *FASN* were analysed for effects on IMF using the same model and haplotype cutoffs. The SNPs in the flanking haplotypes did not include any of the SNPs from the haplotypes that were associated to IMF. The flanking haplotypes were not significantly associated to IMF for the genes *ADIPOQ*, *CAPN1*, *CEBPA*, and the 5′ flanking haplotype of *FASN* or the SNPs adjacent to the *CXCR4* gene. One haplotype of the 3′ flanking haplotype of *FASN*, h121, involving the SNPs ARS-BFGL-NGS-15454, ARS-BFGL-NGS-35888, and Hapmap42556-BTA-45815, was significantly associated to IMF, with an effect of *b* = 0.54, S.E. = 0.20, P = 0.0074, which was of a similar strength to the association for the haplotypes of SNPs that were located over *FASN*.

Haplotype analyses improved the amount of the phenotypic variance explained compared to the amount of variance explained by the individual SNPs or panels of SNPs analysed simultaneously (Table 5). The increase in the phenotypic variance explained by haplotypes compared to individual SNPs was at least 35% for *CEBPA* but larger for other genes. In total, across the 5 genes, the sum of the haplotypes explained 80% more phenotypic variance for the trait than the sum of the best single SNP for each gene. For some genes, such as *CXCR4* and *CEBPA*, which had individual SNPs with 0.01≤*P*<0.001, the amount of variance explained by individual SNPs was almost identical to the amount of variance explained in analyses where only a single, significant, haplotype was fitted, but substantially less than the variance explained when all haplotypes with MHF≥0.05 were fitted. The increase in variance occurred when more than one haplotype was fitted simultaneously. In the single SNP analyses, for *ADIPOQ* and *CXCR4*, the sum of the phenotypic variance explained by the three individual SNPs was larger than the variance explained by fitting all the haplotypes with MHF≥0.05. Counting the variance for three individual SNPs for the same gene could inflate the amount of the variance explained, in those cases where each SNP responded in part to the same variance. The variance explained by fitting three SNPs simultaneously (Table 5) was on average 22.9% less than that found using haplotypes. Nevertheless, because haplotypes take into account the relationship between SNPs, we included the interactions between SNPs. The variance explained by simultaneously fitting three SNPs and their interactions (Table 5) was on average 5.8% less than that found using haplotypes, and for two of the genes, *ADIPOQ* and *CXCR4*, the haplotypes explained less of the phenotypic variance than fitting the three SNPs and their interactions.

**Table 5:** Percent of phenotypic variance for IMF explained by haplotypes compared to SNPs.

	Gene				
Total variance	*ADIPOQ*	*CAPN1*	*CXCR4*	*CEBPA*	*FASN*
3 SNPs summed‡	2.8	0.3	2.0	1.5	1.2
3 SNPs simultaneous	2.2	0.7	1.7	1.5	1.4
3 SNPs plus interactions†	2.6	0.9	1.8	1.6	1.6
3-SNP haplotypes	2.4	1.0	1.7	1.9	1.8

‡ variance of each SNP estimated individually then summed across SNPs.

† simultaneous estimate.

Note that for the gene *CXCR4*, this gene is the closest gene to the significant SNPs, but these are not located within the gene itself.

## Discussion

The results of this study show that an analysis of haplotypes can substantially improve the amount of the phenotypic variance explained compared to single SNPs from a particular region of the genome. Haplotypes explained around 80% more of the phenotypic variance for the five genes that showed some evidence of association to IMF compared to single SNP analyses, suggesting that the amount of variance estimated for GWAS based on single point analyses could be a substantial underestimate of the true variance. Our results show that even if causative mutations are present in the haplotype, single haplotypes based on those causative mutations can explain more variance than the causative mutations. This is contrary to simulation results, which, to be fair, were based on the presence of single causative mutations at genes. Furthermore, haplotypes are neither genetically nor statistically independent observations, so analysing haplotypes in isolation is somewhat artificial, and when the common haplotypes are analysed simultaneously they do explain more variance than single haplotypes alone. With the *CAPN1* gene, it is known that there is more than one QTL segregating for this gene [47], [48], [49], so in this case, the increase in variance explained is due to the effect of the combination of more than one QTL, and this likely applies to other genes as well. Simultaneous fitting of haplotypes is also a more efficient procedure because it 1) avoids the problem of sequential testing which increases the number of tests per genetic region, and 2) provides shrunk estimates of the genetic effects. As most of the SNPs in these genes are not causative, the success of the haplotype analysis in improving the amount of the variance explained suggests that haplotype analyses are a neglected aspect of the genetic analysis of GWAS data. Although we did not explore alternative lengths or SNP content of haplotypes, in the interests of a uniform analysis across several genes, such an approach could certainly be taken where there is *prima facie* evidence that a genetic region was likely to be associated to the trait. Our initial exploration of *CAPN3* suggests that such an approach would indeed be fruitful, as we rediscovered the effect that had been discovered using a different set of SNPs, although the logistics of a GWAS would still militate against running a large number of alternative haplotypes in a region.

We found that simultaneous analyses of the SNPs of the haplotype as well as their interactions can essentially explain a similar amount of the phenotypic variance to that explained by the haplotypes, and which could act as a primary screening tool to determine which regions of the genome should be addressed using an intensive haplotype analysis. Scanning haplotypes across the genome can be difficult given the currently available tools. In this study custom perl scripts were developed to take the output from Beagle, count occurrences of each haplotype for each individual, reformat for ASReml analysis, and then run the ASReml batch job. Although this is computationally time consuming it can be programmed as a batch job. In contrast, it is computationally trivial to run a single point ASReml batch job to do a GWAS, and essentially as simple to run a batch job where windows of a fixed number of SNPs and their interactions could be analysed in performing the GWAS. Combinations of SNPs that explained relatively large amounts of the phenotypic or genetic variance would then be targeted for more in-depth haplotype analysis. This approach would have the added advantage that where two or more SNPs are essentially reporting the same association, only the one with the strongest association will be reported and the others will be knocked down to background levels. Of course, in such analyses it is important to have full data sets, either because the genotypes are complete or because missing data have been imputed. Otherwise, a SNP with a more complete data set but a looser association to the trait might overcome a SNP with an incomplete data set but stronger association to the trait. In our data, even 17 missing data points made the difference between a SNP at *FASN* showing no association (*P*≥0.05) to IMF being upgraded to having some suggestive level (*P* = 0.0049) of association to IMF.

There were no specific characteristics of these haplotypes that increased the rate at which associations were detected, suggesting that detection of an association did not depend on the details of the haplotypes themselves. Although a relatively small number of genes were examined, so subtle effects of haplotypes would not be discovered, there was no clear major effect of haplotype length, LD or other feature which stood out as making one set of haplotypes more likely to find an association than other sets. These haplotypes range from approximately 37 kb to 199 kb in size, and LD ranged from D′ from 0.27 to 1.00 and *r* ^2^ from 0.00 to 0.65 between the outside SNPs of each 3-SNP haplotype. This covers a wide range of different haplotypes and is representative of the Bovine SNP array. In other data sets or arrays, such as the Bovine high density array with 770,000 SNPs, or the various human arrays, haplotypes that have tighter LD relationships could be found, and these might show stronger relationships between the gross physical characteristics of the haplotypes and associations to traits. However, for our data set, the lack of a strong effect of these gross characteristics suggests that imponderables such as which SNP to choose for the haplotype or the exact LD relationships between SNPs is of lesser importance than whether a causative allele is present and whether the sample is large enough to detect the effect of that causative mutation.

Haplotype analysis did not detect associations to all the candidate genes, although one would not have expected associations to all these candidate genes. Firstly, some of the SNPs were for non-IMF muscle fat traits, either marbling score (MS) or percent saturated fat (PSF), and not IMF itself. Although MS is dependent on IMF for its expression, MS and IMF are correlated with *r*<0.5 [51]. In addition, PSF and MS are loosely correlated, because the speed with which MS develops post-mortem is affected by the degree of saturation of fatty acids, but the effect is not strong. IMF and PSF are not directly related, but most dietary fatty acids in cattle are saturated due to the action of bacteria in the rumen, and food composition has a larger effect on PSF than the genetic differences between animals [52]. These relationships suggest that genetic associations to IMF for some of these candidate genes could be substantially different [53] to the original traits, but with some possibility of overlap. Secondly, for genes such as *LEP* and *TG*, the QTLs tagged by the specific DNA markers in these genes do not appear to be segregating in the Beef CRC sample [54], [55]. Thirdly, for genes such as *CPE* and *RORC*, which have been detected in the Beef CRC resource, either in smaller samples (*CPE*) or as a small effect in much larger samples (*RORC*) [56], [57], the lack of association may point to insufficient LD between the SNPs or their haplotypes, or insufficient power, in this study.

Nevertheless, this study reports the first confirmation of an effect of *ADIPOQ* on IMF as well as the first time that *FASN* has been reported to have an effect on IMF. In the single point analysis, *ADIPOQ* was not strongly supported because 2 of the SNPs have very low MAF, but once haplotypes were used, the combined data became more powerful to detect the effect. *FASN* has been reported to have an effect on milk fat percentage (MFP) in dairy cows [58] and PSF [59], [60], [61], [62] but this is the first association to IMF. Although the association to PSF is well established, an association to IMF and MFP is more consistent with the role of the gene, which is to construct long chain saturated fatty acids (i.e., palmitic acid) from shorter chain precursors [63], most of which are saturated in cattle due to microbial action on fatty acids in the rumen [64], and only delta-9 desaturase (stearoyl-CoA desaturase *SCD*) converts saturated fat to unsaturated fat in cattle [52]. This suggests that the effect of *FASN* on PSF is primarily due to its action on the saturated fraction of total fatty acids. This study also found suggestive evidence for haplotypes of *CEBPA* associated with IMF, although tests performed using a similar sized sample of the Beef CRC data and the published test had failed to find an association at the *P* = 0.05 threshold [56]. Finally, associations between haplotypes for *CXCR4* and IMF were also identified. This region had previously been identified through the intersection of population genetic evidence of selection and QTL analyses [65] but when that analysis was performed the state of the assembly of the bovine genome pointed to a region without genes near to the gene *R3HDM1*. The 3 SNPs near *R3HDM1* had been included in this study as an example of a set of confirmed SNPs that were not associated to a candidate gene. However, improvements in the bovine assembly showed that the SNPs used in this study are adjacent to the gene *CXCR4*, a gene with several known effects including vascularisation of organs [66]. IMF is laid down along capillary beds in muscle [67], so *CXCR4* could be considered a legitimate candidate gene for MS and IMF by making available sites for the deposition of fat.

In conclusion, our results show that haplotype analysis of GWAS data should not be neglected, that in some examples it provides substantially more variance than single SNP analysis, and that preliminary analysis using simultaneously fitted groups of SNPs and their interactions is a convenient shortcut to identify regions that are worth analysing in detail using haplotypes. What is not yet clear is how decisions should be made on the number and identity of SNPs to be included in the haplotypes. Some of the questions are: is it worth dropping some SNPs, should SNPs always be in groups of adjacent SNPs, and how open ended should haplotype analyses be in exploring which haplotypes explain the most variation in the data. Our data are still not dense enough to explore this in more detail, and data sets that essentially represent the bulk of the SNPs of a gene would be a useful place to start.

## Materials and Methods

Animal Care and Use Committee approval was not obtained for this study because no new animals were handled in this experiment. The analysis was performed on trait records, DNA samples and genotypes that had been collected previously. The animals in this experiment were born between 1993 and 1999 as described below.

Cattle consist of two subspecies, the taurine breeds of *Bos taurus taurus*, and the indicine breeds of *B. taurus indicus*. These subspecies are fully inter-fertile and show heterosis in the first generation cross. Stable composites of the two subspecies have been bred over many generations. The taurine breeds were Angus (ANG), Hereford (HFD), Murray Grey (MGY), and Shorthorn (SHN), the indicine breed was Brahman (BRM) and the stable composites were Belmont Red (BEL), and Santa Gertrudis (SGT). The animals in the BEL and SGT samples have all 4 grandparents as registered stud animals for those breeds and are not of recent crossbred origin. Such animals are treated as purebred for the purposes of this analysis.

The breeding and measurement of IMF and LLPF of these 940 beef cattle of the Genetic Correlations Experiment of the Cooperative Research Centre for the Cattle and Beef Industry (Beef CRC) was reported previously [68], [69]. A summary of the raw phenotypes for IMF and LLPF for the subsample of 940 animals used in this study is shown in Tables S1 and S2. The breed composition of the sample consisted of 220 ANG, 146 HFD, 55 MGY, 81 SHN, 78 BRM, 165 BEL, 126 SGT, 25 Taurine-Brahman and 44 Composite-Brahman first generation crossbred animals. These represent the offspring of 246 sires, and 34 herds of origin, each breed consisting of several herds of origin, two sexes, and 50 measurement days. The average number of half-sibs per sire was 3.8 with a range of 1 to 15 offspring per sire. The genotypes for these animals were reported previously [44]. In brief, genotypes for 53,798 SNPs were available for these animals from an Illumina Bovine SNP50 v1 array [70]. The data were exported as AA, AB and BB genotypes in the Illumina top/bot format. For single locus regression analyses, the genotypes were recoded as 0, 1, and 2 B alleles.

The genotypes for each SNP in this study were analysed for departures from Hardy-Weinberg Equilibrium (HWE) within breed, LD between SNPs in the same region in each breed was estimated using D′ [71], and *r* ^2^ [72] corrected by subtracting the reciprocal of the sample size [73]. *r*, the square root of *r* ^2^, was examined to determine whether the same alleles where part of common haplotypes [72]. Mean LD values were calculated per breed and were not estimated from animals from a mixture of breeds. The difference in genotype and haplotype counts between breeds was determined using the log likelihood test with the Williams correction [74], [75]. F_ST_ between breeds was calculated for each SNP using the Weir and Cockerham method [65], [76].

For haplotype analysis, the data were ordered by position along each chromosome using the Btau4.0 and UMD3.1 assemblies [77], [78], haplotype phase was then inferred and missing data imputed using BEAGLE version 3.3.1 [79]. In the estimation of phase, data were stratified by breed but were treated as unrelated because the dataset did not consist of parent offspring trios. The phase determination was iterated 20 times, was performed in windows of 500 adjacent SNPs, and was analysed a chromosome at a time for the autosomes only. Given the spacing of SNPs (∼50 kb between adjacent SNPs) and the size of genes, in all cases 3 adjacent SNPs were combined to form a haplotype. In some cases the gene was significantly smaller than the haplotype of 3 SNPs, but in some cases the gene extended well beyond the confines of the haplotype. Except for *CXCR4*, the central SNP was placed as close to the coding sequence of the gene as possible, and where the gene was larger than the haplotype, the 5′ region of the gene was targeted. The same number of SNPs was used for all genes to facilitate comparisons between genes and to overcome some of the arbitrary nature of haplotype analysis, namely, how many SNPs should be included in the haplotype, should the haplotype consist of adjacent SNPs only, and the sequential testing of a wide range of haplotypes to discover the best haplotype for the region, a process that always generates a large number of comparisons. The number of copies of each haplotype was counted for each animal, leading to a vector of 0 s, 1 s, and 2 s for each animal that was equal in length to the number of haplotypes at the gene. Given that haplotype phase and missing data imputation is most accurate with common alleles [80], rare haplotypes, those with MHF<0.05, were excluded from the association analysis. For all analyses, all haplotypes with MHF≥0.05 were fitted simultaneously in the regression analysis because haplotypes are not independent, that is, if all *n* haplotypes are fitted simultaneously then only *n*−1 partial haplotype substitution effects can be estimated. In some examples, to illustrate some of the data that can be obtained from haplotypes, subsets of the haplotypes were analysed.

The phenotypes and genotypes or haplotypes were fitted in a restricted (or residual) maximum likelihood (REML) mixed model of the form trait∼mean+fixed effects+genotypes+animal+error using the software ASReml v3.0 [81] where animal and error were random effects and genotypes was either a variable consisting of the number of copies of an allele or consisted of all the common haplotypes fitted simultaneously, in a REML process analogous to a type III ANOVA. Allele substitution effects or the partial haplotype substitution effects were evaluated through a t-test based on the allele or partial haplotype substitution effect divided by its standard error. The fixed effects were breed, herd of origin, sex, and date of measurement [44]. Age on day of measurement was added as a covariate. Relationships between individuals were evaluated using a numerator matrix derived from five generations worth of pedigree information. There were several herds within each breed, and herd of origin was fitted in case there were allele frequency differences between herds within a breed. Heritability estimates and their standard errors were obtained from these models. In the original GWAS, multiple testing was accommodated using a False Discovery Rate model and SNPs were identified for further testing if the significance of the association was generally *P*<0.001, although a set of SNPs with *P*<0.005 was also tested to determine whether the threshold made a difference to the number of successfully confirmed SNP associations. In this current study we compared analyses of single point associations of SNPs of candidate genes chosen for *a priori* reasons to analyses using haplotypes of the SNPs at the same candidate gene, the haplotypes analysed simultaneously, so issues of correction of multiple testing are not particularly relevant. Of more relevance is the *R* ^2^ or variance [22], [82] explained using single point analyses versus the effect estimated using haplotypes. Here, the overall proportion of the phenotypic variance (*R* ^2^) of the simultaneously fitted haplotypes was estimated by comparing the residual sums of squares (RSS) of a model with haplotypes (RSS_w_) to the RSS of a model without haplotypes (RSS_n_) using the equation
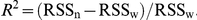
The RSS contained the variability due to the pedigree as well as the fixed, random, and error terms and so is an estimate of the total phenotypic variance. The same method was applied for genotypes of SNPs fitted singly or as a group of 3 SNPs fitted simultaneously, using imputed genotypes, to allow comparison to the estimates from haplotypes.

To analyse genes, their locations, and the position of SNPs on the map, the Btau 4.0 and UMD3.1 Bovine Genome Assemblies implemented at http://www.livstockgenomics.csiro.au/perl/gbrowse.cgi/bova4/ [77], [78] were used. SNPs in genes that had previously been associated with intramuscular fat in some way, whether as MS, PSF, or IMF, were tested to see whether any were significantly (*P*<0.05) associated to IMF in the GWAS study. Previous studies had found associations between MS, PSF, or IMF, and SNPs in the alphabetically listed candidate and positional candidate genes adiponectin, C1Q and collagen domain containing (*ADIPOQ*) [83], calpain 1 (*CAPN1*) [84], carboxypeptidase E (*CPE*) [56], [85], CCAAT/enhancer binding protein (C/EBP) alpha (*CEBPA*) [86], the region containing the chemokine (C-X-C) motif receptor 4 (*CXCR4*) gene near to the genes R3H domain containing 1 (*R3HDM1*) and zinc finger, RAN domain containing 3 (*ZRANB3*) [65], 2,4 dienoyl CoA reductase 1 (*DECR1*) [87], fatty acid binding protein 4 (*FABP4*) [88], fatty acid synthase (*FASN*) [59], fibroblast growth factor 8 (*FGF8*) [87], growth hormone 1 (*GH1*) [89], [90], growth hormone receptor (*GHR*) [91], insulin growth factor 2 (*IGF2*) [92], leptin (*LEP*) [93], retinoic acid receptor-related orphan receptor C (*RORC*) [94], sterol regulatory element binding transcription factor 1 (*SREBF1*) [95], steroyl-CoA desaturase (*SCD*) [96], thyroglobulin (*TG*) [97], and titin-cap (telethonin) (*TCAP*) [98]. For the trait LLPF, SNPs in the candidate genes *CAST*, *CAPN1* and calpain 3 (*CAPN3*) have been reported in cattle [46], [47], [48], [49], [50]. Improvements in the bovine assembly have identified that the SNPs associated to IMF near *R3HDM1* and *ZRANB3* flank the gene *CXCR4*, although they are not in *CXCR4* itself. Apart from the CAPN1_1 and CAPN1_2 SNP, that were associated to LLPF in previous studies, none of the SNPs that were previously associated to any of these traits was part of the Bovine SNP50 array. Nevertheless, the regions containing these genes do have SNPs represented on the SNP50 array, so through LD it may be possible to evaluate some of the effects of these genes.

## Supporting Information

Table S1 Uncorrected IMF variability in the different breeds.
(PDF)

Table S2 Uncorrected LLPF variability in the different breeds.
(PDF)

Table S3 Allele frequencies, alternative alleles, sample sizes, and genomic locations for each of the SNPs in the study.
(XLS)

Table S4 Haplotype counts for each gene in each pure breed.
(PDF)

Table S5 Size of haplotype, linkage disequilibrium measures, and haplotype consistency for haplotypes of each gene.
(XLS)

Table S6 Single point SNP associations of candidate genes for IMF in the GWAS.
(PDF)

Table S7 Haplotype associations of candidate genes for IMF in the GWAS.
(PDF)
