# Supplementary material for: Haplotype Analysis Improved Evidence for Candidate Genes for Intramuscular Fat Percentage from a Genome Wide Association Study of Cattle
Source: PLoS One. 2011 Dec 28;6(12):e29601. doi: 10.1371/journal.pone.0029601 (PMC3247274; doi:10.1371/journal.pone.0029601)
Supplement: Table S1 — Uncorrected IMF variability in the different breeds. (PDF) [file pone.0029601.s001.pdf]

Haplotype analysis improved evidence for candidate genes for intramuscular fat percentage from a genome wide association study of cattle

W. Barendse

Supplementary Tables

Table S1. Uncorrected IMF variability in the different breeds

Breed	n	x̄ (%)	s.d.	min	max	CV
Angus	220	6.1	2.2	2.0	12.0	0.36
Hereford	146	4.7	1.6	2.0	10.1	0.35
Murray Grey	55	5.8	2.1	2.0	11.5	0.36
Shorthorn	81	5.8	1.9	2.2	11.7	0.33
Belmont Red	165	3.9	1.6	1.5	10.6	0.40
Santa Gertrudis	126	3.8	1.5	1.4	10.6	0.39
Angus x Brahman	11	5.0	2.0	2.7	9.2	0.40
Hereford x Brahman	8	4.5	1.3	3.1	6.7	0.28
Shorthorn x Brahman	6	3.2	0.2	3.0	3.6	0.06
Belmont Red x Brahman	35	3.4	1.1	2.1	6.4	0.33
Santa Gertrudis x Brahman	9	3.1	0.9	2.0	4.7	0.30
Brahman	78	3.2	1.8	1.4	12.6	0.57
Total	940	4.7	2.1	1.4	12.6	0.44
