# Supplementary material for: Haplotype Analysis Improved Evidence for Candidate Genes for Intramuscular Fat Percentage from a Genome Wide Association Study of Cattle
Source: PLoS One. 2011 Dec 28;6(12):e29601. doi: 10.1371/journal.pone.0029601 (PMC3247274; doi:10.1371/journal.pone.0029601)
Supplement: Table S2 — Uncorrected LLPF variability in the different breeds. (PDF) [file pone.0029601.s002.pdf]

Haplotype analysis improved evidence for candidate genes for intramuscular fat percentage from a genome wide association study of cattle

W. Barendse

Supplementary Tables

Table S2. Uncorrected LLPF variability in the different breeds

Breed	n	$\bar{x}$ (%)	s.d.	min	max	CV
Angus	220	3.9	0.6	2.7	6.2	0.14
Hereford	144	4.0	0.8	2.5	8.2	0.19
Murray Grey	55	3.8	0.5	2.4	5.2	0.14
Shorthorn	81	4.3	0.9	2.8	7.2	0.20
Belmont Red	165	4.4	0.9	3.2	9.7	0.21
Santa Gertrudis	125	4.4	0.9	2.4	7.7	0.21
Angus x Brahman	11	4.2	0.6	3.3	5.1	0.14
Hereford x Brahman	8	4.2	0.3	3.8	4.6	0.07
Shorthorn x Brahman	6	4.7	0.9	4.2	5.3	0.09
Belmont Red x Brahman	35	4.6	0.6	3.7	6.6	0.14
Santa Gertrudis x Brahman	9	4.2	0.5	3.3	5.0	0.12
Brahman	77	5.1	1.1	3.0	9.7	0.21
Total	936	4.2	0.9	2.4	9.7	0.21
