# Supplementary material for: Haplotype Analysis Improved Evidence for Candidate Genes for Intramuscular Fat Percentage from a Genome Wide Association Study of Cattle
Source: PLoS One. 2011 Dec 28;6(12):e29601. doi: 10.1371/journal.pone.0029601 (PMC3247274; doi:10.1371/journal.pone.0029601)
Supplement: Table S4 — Haplotype counts for each gene in each pure breed. (PDF) [file pone.0029601.s004.pdf]

Haplotype analysis improved evidence for candidate genes for intramuscular fat percentage from a genome wide association study of cattle

W. Barendse

Supplementary Tables

Table S4. Haplotype counts for each gene in each pure breed

		Haplotypes							
Gene	Breed	111	112	121	122	211	212	221	222
ADIPOQ	ANG	0	0	0	356	0	1	0	83
	HFD	0	0	28	245	10	2	0	7
	MGY	0	0	0	103	0	0	0	7
	SHN	0	0	0	153	0	0	0	9
	BEL	0	0	1	169	0	5	0	155
	SGT	0	0	0	184	0	0	0	68
	BRM	0	0	0	39	0	0	0	117
CAPN1	ANG	0	175	62	129	0	0	36	38
	HFD	9	52	25	135	0	0	13	58
	MGY	0	48	0	22	0	0	26	14
	SHN	0	0	38	3	0	0	121	0
	BEL	5	139	109	29	0	0	20	28
	SGT	39	27	101	61	0	0	17	7
	BRM	2	2	127	16	0	1	5	3
CAPN3	ANG	2	0	152	0	236	0	6	44
	HFD	0	0	90	0	68	0	134	0
	MGY	0	0	19	0	43	0	27	21
	SHN	0	0	60	0	59	0	35	8
	BEL	11	0	54	14	101	0	141	9
	SGT	5	0	41	51	61	0	81	13
	BRM	0	0	2	47	3	0	73	31
CAST	ANG	0	0	58	85	297	0	0	0
	HFD	0	0	55	12	225	0	0	0
	MGY	0	0	15	48	47	0	0	0
	SHN	0	0	111	0	50	1	0	0
	BEL	0	0	9	60	203	16	4	38
	SGT	0	1	8	52	121	29	22	19
	BRM	1	0	1	1	43	33	9	68
CEBPA	ANG	0	0	12	0	122	286	0	20
	HFD	0	0	19	0	3	184	25	61
	MGY	0	0	7	0	2	93	0	8
	SHN	0	0	0	0	6	124	1	31
	BEL	0	0	11	29	3	103	1	183
	SGT	0	0	0	0	1	180	0	71
	BRM	0	0	0	18	0	12	5	121
CPE	ANG	98	149	4	129	12	48	0	0
	HFD	0	176	49	65	1	1	0	0

	MGY	55	14	1	11	0	20	0	9
	SHN	8	48	1	49	0	6	0	50
	BEL	1	135	13	106	2	51	5	17
	SGT	2	71	2	68	2	83	0	24
	BRM	2	4	4	21	5	80	2	38
CRH	ANG	0	0	1	62	0	86	118	173
	HFD	0	0	0	82	0	141	4	65
	MGY	0	0	0	5	0	49	32	24
	SHN	0	0	0	90	0	14	14	44
	BEL	0	8	55	135	0	67	12	53
	SGT	0	3	23	97	0	88	15	26
	BRM	0	4	18	33	0	40	10	51
CXCR4	ANG	0	0	0	1	0	25	216	198
	HFD	0	0	0	0	0	89	14	189
	MGY	0	0	0	0	0	5	56	49
	SHN	0	0	0	0	0	1	12	149
	BEL	0	0	0	17	0	11	21	281
	SGT	0	0	0	3	0	54	11	184
	BRM	0	0	0	6	0	8	12	130
DECR1	ANG	38	68	1	1	5	0	282	45
	HFD	28	41	0	0	75	29	18	101
	MGY	13	14	0	0	2	0	52	29
	SHN	1	50	0	6	23	5	54	23
	BEL	48	93	11	0	49	11	96	22
	SGT	34	175	0	2	13	1	21	6
	BRM	72	69	0	0	1	0	6	8
DGAT1	ANG	0	0	0	0	0	428	0	12
	HFD	0	0	0	0	29	263	0	0
	MGY	0	0	0	0	0	104	0	6
	SHN	0	0	0	0	0	162	0	0
	BEL	0	0	0	0	7	199	0	124
	SGT	0	0	0	0	1	130	0	121
	BRM	0	0	0	1	0	27	0	128
FABP4	ANG	0	0	0	83	22	74	210	51
	HFD	0	0	7	39	71	22	108	45
	MGY	0	0	0	39	5	36	15	15
	SHN	0	1	44	22	1	72	10	12
	BEL	0	9	59	28	35	110	36	53
	SGT	0	3	48	27	10	72	12	80
	BRM	0	31	2	7	8	80	16	12
FASN	ANG	0	117	6	1	27	184	104	1
	HFD	0	0	50	0	0	142	48	52
	MGY	0	26	1	0	12	30	30	11
	SHN	0	1	0	0	8	97	37	19
	BEL	0	0	18	0	5	30	207	70
	SGT	0	0	2	0	1	26	190	33
	BRM	0	0	2	0	0	0	149	5
FGF8	ANG	84	16	224	0	116	0	0	0

	HFD	210	29	32	0	21	0	0	0
	MGY	0	0	69	0	41	0	0	0
	SHN	115	26	10	0	10	0	1	0
	BEL	266	8	11	0	36	1	8	0
	SGT	187	27	29	0	9	0	0	0
	BRM	142	9	3	0	2	0	0	0
GH1	ANG	38	150	54	89	28	2	79	0
	HFD	60	67	33	2	3	16	109	2
	MGY	6	67	7	14	14	0	2	0
	SHN	16	2	45	63	35	0	0	1
	BEL	67	13	3	20	19	6	18	184
	SGT	7	26	26	86	12	1	9	85
	BRM	4	25	2	27	8	2	21	67
GHR	ANG	31	63	0	294	0	52	0	0
	HFD	104	0	0	140	0	37	0	11
	MGY	0	28	0	63	0	19	0	0
	SHN	14	12	0	104	0	25	0	7
	BEL	12	2	0	301	0	15	0	0
	SGT	12	1	0	162	0	77	0	0
	BRM	3	0	0	148	0	5	0	0
IGF2	ANG	0	0	6	268	1	85	0	80
	HFD	0	0	47	132	0	11	20	82
	MGY	0	0	0	56	0	20	13	21
	SHN	0	0	4	100	0	2	0	56
	BEL	0	0	4	57	41	14	9	205
	SGT	0	0	19	128	1	19	1	84
	BRM	0	0	0	13	0	13	1	129
LEP	ANG	1	196	23	0	0	59	149	12
	HFD	0	114	0	19	0	126	33	0
	MGY	0	14	19	1	0	23	53	0
	SHN	2	37	1	1	0	116	5	0
	BEL	0	170	7	21	0	103	20	9
	SGT	1	148	2	26	0	73	2	0
	BRM	0	38	9	89	0	14	6	0
RORC	ANG	0	0	0	121	0	95	117	107
	HFD	0	0	0	0	47	214	26	5
	MGY	0	0	0	46	0	38	6	20
	SHN	0	0	0	50	1	104	4	3
	BEL	0	0	0	10	19	134	25	142
	SGT	0	0	0	58	2	103	21	68
	BRM	0	0	0	2	1	7	50	96
SCD	ANG	0	1	0	0	0	133	44	262
	HFD	0	0	0	53	0	0	6	233
	MGY	0	0	0	0	0	12	20	78
	SHN	0	0	0	4	0	32	0	126
	BEL	0	0	1	51	0	35	0	243
	SGT	0	0	0	14	0	38	4	196
	BRM	0	0	0	18	0	15	0	123

SREBF1	ANG	208	0	0	0	50	2	2	178
	HFD	112	2	0	0	31	0	0	147
	MGY	29	0	0	0	0	0	2	79
	SHN	84	0	0	0	1	32	0	45
	BEL	27	0	0	0	14	7	1	281
	SGT	42	0	0	0	3	8	0	199
	BRM	10	0	0	0	1	0	0	145
TCAP	ANG	0	337	79	11	12	1	0	0
	HFD	5	179	44	0	64	0	0	0
	MGY	1	48	45	16	0	0	0	0
	SHN	0	122	1	39	0	0	0	0
	BEL	29	121	53	6	102	0	19	0
	SGT	1	136	1	64	36	0	14	0
	BRM	1	10	4	2	60	0	79	0
TG	ANG	0	112	184	0	17	127	0	0
	HFD	0	104	31	13	0	52	86	6
	MGY	0	32	21	0	0	57	0	0
	SHN	0	64	86	3	0	7	2	0
	BEL	0	43	52	4	0	154	17	60
	SGT	0	43	89	1	1	14	12	92
	BRM	0	4	10	0	0	15	3	124

Note that for the gene *CXCR4*, this gene is the closest gene to the significant SNPs, but these are not located within the gene itself.
