# Supplementary material for: Haplotype Analysis Improved Evidence for Candidate Genes for Intramuscular Fat Percentage from a Genome Wide Association Study of Cattle
Source: PLoS One. 2011 Dec 28;6(12):e29601. doi: 10.1371/journal.pone.0029601 (PMC3247274; doi:10.1371/journal.pone.0029601)
Supplement: Table S6 — Single point SNP associations of candidate genes for IMF in the GWAS. (PDF) [file pone.0029601.s006.pdf]

Haplotype analysis improved evidence for candidate genes for intramuscular fat percentage from a genome wide association study of cattle

W. Barendse

Supplementary Tables

Table S6. Single point SNP associations of candidate genes for IMF in the GWAS

SNP	A	B	Bta	Position	*b* (%)	s.e.	*P*
**ADIPOQ**							
ARS-BFGL-NGS-26946	A	G	1	82201457	0.049	0.103	0.6316
Hapmap43250-BTA-37524	A	G	1	82245379	-0.956	0.378	0.0117
BTB-00035080	A	G	1	82271202	-0.642	0.274	0.0191
**CXCR4**							
ARS-BFGL-NGS-117383	A	G	2	63905821	0.331	0.413	0.4239
Hapmap55796-rs29011172	A	T	2	63947669	-0.427	0.135	0.0016
ARS-BFGL-NGS-119079	A	G	2	63998173	0.297	0.116	0.0107
**RORC**							
ARS-BFGL-NGS-52272	C	G	3	20421496	0.217	0.115	0.0588
INRA-404	A	G	3	20519554	-0.128	0.092	0.1640
ARS-BFGL-NGS-109685	A	G	3	20564445	0.101	0.110	0.3568
**LEP**							
ARS-BFGL-NGS-101028	A	G	4	95639597	-0.033	0.088	0.7084
ARS-BFGL-NGS-59298	A	G	4	95685551	-0.065	0.099	0.5121
ARS-BFGL-NGS-34894	A	G	4	95715499	0.160	0.116	0.1673
**CAST**							
ARS-BFGL-NGS-43901	A	C	7	97492911	-0.021	0.092	0.8231
USMARC-670	A	G	7	97524770	-0.040	0.084	0.6316
USMARC-116	A	G	7	97561407	-0.005	0.093	1.0000
**CAPN3**							
ARS-BFGL-NGS-13350	A	G	10	37625930	0.004	0.087	1.0000
Hapmap47063-BTA-62293	A	G	10	37647411	-0.097	0.087	0.2620
ARS-BFGL-BAC-12264	A	G	10	37675399	-0.332	0.129	0.0101
**DGAT1**							
ARS-BFGL-BAC-21527	A	C	14	399173	-0.679	1.650	0.6802
ARS-BFGL-NGS-4939	A	G	14	443937	-0.137	0.125	0.2736
ARS-BFGL-BAC-26048	A	G	14	483500	0.060	0.309	0.8415
**TG**							
Hapmap36763-SCAFFOLD240007_5847	A	C	14	7620648	-0.011	0.090	0.8876
ARS-BFGL-NGS-11246	A	G	14	7656719	0.061	0.090	0.5025
ARS-BFGL-NGS-12631	A	G	14	7685715	-0.029	0.090	0.7402
**CRH**							

ARS-BFGL-NGS-70865	A	G	14	30425753	-0.121	0.094	0.1966
ARS-BFGL-NGS-25050	A	G	14	30447263	0.042	0.094	0.6548
ARS-BFGL-NGS-26147	A	G	14	31515559	0.126	0.108	0.2439
**FABP4**							
BTB-00567254	A	G	14	41915068	-0.057	0.094	0.5432
BTB-00567299	A	G	14	41966954	0.123	0.089	0.1684
BTB-00567509	A	G	14	42054159	-0.142	0.087	0.1026
**DECR1**							
ARS-BFGL-BAC-23724	A	G	14	72289416	0.030	0.096	0.7519
UA-IFASA-7973	A	G	14	72368404	0.051	0.095	0.5904
BTA-35525-no-rs	A	G	14	72400485	-0.028	0.085	0.7402
**CPE**							
Hapmap54135-rs29019936	A	G	17	573316	0.113	0.101	0.2658
ARS-BFGL-NGS-111865	A	G	17	657048	0.136	0.083	0.1013
Hapmap41213-BTA-26249	A	G	17	771833	0.088	0.117	0.4505
**CEBPA**							
ARS-BFGL-NGS-105692	A	G	18	43119331	-0.076	0.176	0.6715
ARS-BFGL-NGS-21339	A	G	18	43150185	0.265	0.101	0.0092
BTA-43268-no-rs	A	G	18	43170819	-0.188	0.123	0.1273
**SREBF1**							
ARS-BFGL-NGS-111809	A	G	19	35666624	-0.140	0.102	0.1707
ARS-BFGL-NGS-4759	A	G	19	35690367	-0.046	0.092	0.6172
ARS-BFGL-NGS-23022	A	G	19	35723058	-0.106	0.091	0.2421
**TCAP**							
Hapmap49048-BTA-119203	A	G	19	41230750	0.027	0.122	0.8231
ARS-BFGL-NGS-57711	A	G	19	41301795	-0.113	0.093	0.2273
ARS-BFGL-NGS-32460	A	G	19	41325600	-0.049	0.098	0.6243
**GH1**							
ARS-BFGL-NGS-78203	A	C	19	49617252	0.003	0.086	1.0000
ARS-BFGL-NGS-73805	A	G	19	49652376	0.035	0.089	0.6893
ARS-BFGL-NGS-115719	A	G	19	49690249	-0.052	0.086	0.5431
**FASN**							
ARS-BFGL-NGS-20701	A	C	19	52158614	-0.185	0.125	0.1393
ARS-BFGL-NGS-39983	A	G	19	52182484	0.133	0.102	0.1940
ARS-BFGL-NGS-73980	A	G	19	52291308	-0.077	0.088	0.3805
**GHR**							
UA-IFASA-8974	A	C	20	33908596	0.070	0.120	0.5600
ARS-BFGL-NGS-118998	A	G	20	34036832	-0.076	0.090	0.3997
ARS-BFGL-NGS-97963	A	G	20	34080607	0.187	0.140	0.1825
**SCD**							
Hapmap31825-BTA-158647	A	G	26	21476708	0.149	0.143	0.2968
Hapmap33073-BTA-162864	A	G	26	21586449	-0.070	0.115	0.5432
BTB-00931481	A	G	26	21631981	0.012	0.212	1.0000

**FGF8**							
Hapmap53405-rs29014382	A	G	26	22772482	0.027	0.116	0.8066
ARS-BFGL-NGS-29678	A	C	26	22816416	0.115	0.109	0.2946
BTB-00933928	A	G	26	22878425	-0.013	0.164	0.9204
**CAPN1**							
ARS-BFGL-NGS-21416	A	G	29	45202710	-0.019	0.104	0.8625
CAPN1_1	C	G	29	45221190	-0.192	0.091	0.0348
CAPN1_2	A	G	29	45239821	-0.008	0.099	0.9204
**IGF2**							
ARS-BFGL-NGS-106809	A	G	29	51231973	-0.111	0.086	0.1979
UA-IFASA-1391	A	G	29	51257883	0.064	0.127	0.6172
ARS-BFGL-NGS-29984	A	G	29	51301848	0.151	0.144	0.2968

Note that for the gene *CXCR4*, this gene is the closest gene to the significant SNPs, but these are not located within the gene itself.
