# Supplementary material for: Haplotype Analysis Improved Evidence for Candidate Genes for Intramuscular Fat Percentage from a Genome Wide Association Study of Cattle
Source: PLoS One. 2011 Dec 28;6(12):e29601. doi: 10.1371/journal.pone.0029601 (PMC3247274; doi:10.1371/journal.pone.0029601)
Supplement: Table S7 — Haplotype associations of candidate genes for IMF in the GWAS. (PDF) [file pone.0029601.s007.pdf]

Haplotype analysis improved evidence for candidate genes for intramuscular fat percentage from a genome wide association study of cattle

W. Barendse

Supplementary Tables

Table S7. Haplotype associations of candidate genes for IMF in the GWAS

Haplotype	*b* (%)	s.e.	*P*
**ADIPOQ**			
h222	-0.985	0.238	3.8e-05
h122	-0.879	0.224	9.2e-05
**CXCR4**			
h222	-0.307	0.120	0.0105
h221	-0.518	0.148	5.0e-04
h122	-0.485	0.291	0.0960
or			
h212	0.337	0.119	0.0048
**RORC**			
h222	0.180	0.200	0.3677
h221	0.064	0.207	0.7593
h212	0.179	0.189	0.3415
h122	0.035	0.209	0.8657
**LEP**			
h221	0.114	0.196	0.5616
h212	0.168	0.175	0.3370
h122	0.402	0.205	0.0506
h112	0.220	0.168	0.1908
**CAST**			
h222	-0.079	0.119	0.5073
h212	-0.100	0.097	0.2987
h211	0.261	0.250	0.2960
h122	0.110	0.122	0.3680
h121	0.151	0.176	0.3885
**CAPN3**			
h222	-0.154	0.362	0.6711
h221	0.005	0.340	0.9874
h211	0.106	0.342	0.7558
h122	-0.010	0.363	0.9774
h121	0.065	0.341	0.8490
**DGAT1**			
h222	-0.053	0.104	0.6077
h122	0.280	1.050	0.7901
**TG**			
h222	-0.128	0.270	0.6360
h221	-0.257	0.283	0.3635

h212	-0.067	0.253	0.7902
h121	-0.061	0.252	0.8085
h112	-0.188	0.251	0.4537
**CRH**			
h222	0.092	0.100	0.3610
h221	-0.018	0.130	0.8892
h122	0.124	0.098	0.2053
h121	0.085	0.166	0.6097
**FABP4**			
h222	-0.060	0.236	0.8001
h221	0.008	0.236	0.9715
h212	-0.137	0.222	0.5382
h211	-0.018	0.252	0.9435
h122	-0.142	0.240	0.5550
h121	0.029	0.262	0.9119
**DECR1**			
h222	0.227	0.205	0.2690
h221	0.078	0.195	0.6897
h211	0.104	0.206	0.6123
h112	0.066	0.193	0.7347
h111	0.079	0.210	0.7083
**CPE**			
h222	0.099	0.198	0.6157
h212	-0.141	0.172	0.4133
h122	-0.043	0.159	0.7871
h112	-0.151	0.152	0.3211
h111	-0.193	0.199	0.3341
**CEBPA**			
h221	0.192	0.284	0.4997
h212	-0.292	0.097	0.0028
h211	-0.126	0.163	0.4413
h122	0.026	0.212	0.9007
h121	-0.374	0.231	0.1054
or			
h212	-0.236	0.078	0.0027
**SREBF1**			
h222	0.354	0.205	0.0840
h211	0.443	0.252	0.0786
**TCAP**			
h221	-0.168	0.283	0.5527
h211	-0.150	0.251	0.5515
h122	-0.455	0.270	0.0920
h121	0.004	0.250	0.9866
h112	-0.148	0.241	0.5395
**GH1**			
h222	-0.109	0.303	0.7198
h221	-0.123	0.301	0.6840
h211	-0.188	0.325	0.5631

h122	-0.103	0.310	0.7398
h121	-0.061	0.316	0.8476
h112	-0.237	0.301	0.4315
h111	0.000	0.307	0.9994
**FASN**			
h222	0.302	0.224	0.1781
h221	0.224	0.205	0.2737
h212	0.051	0.205	0.8018
h121	0.338	0.264	0.2004
h112	0.519	0.229	0.0238
or			
h221	-0.102	0.091	0.2623
h212	-0.287	0.100	0.0042
or			
h212	-0.239	0.090	0.0081
**GHR**			
h212	0.481	0.373	0.1976
h122	0.419	0.365	0.2517
h112	0.607	0.388	0.1185
h111	0.360	0.372	0.3333
**SCD**			
h222	-0.083	0.117	0.4776
h212	0.204	0.142	0.1521
h122	-0.169	0.195	0.3843
**FGF8**			
h211	-0.405	0.439	0.3567
h121	-0.521	0.440	0.2361
h112	-0.428	0.452	0.3441
h111	-0.485	0.431	0.2602
**CAPN1**			
h222	0.168	0.232	0.4674
h221	0.156	0.230	0.4985
h122	-0.102	0.208	0.6230
h121	0.080	0.203	0.6936
h112	0.186	0.210	0.3762
or			
h222	0.003	0.135	0.9847
h221	-0.007	0.138	0.9564
h122	-0.263	0.102	0.0100
h121	-0.074	0.102	0.4684
**IGF2**			
h222	0.042	0.133	0.7510
h212	-0.000	0.170	0.9982
h122	0.126	0.135	0.3505

Note that for the gene *CXCR4*, this gene is the closest gene to the significant SNPs, but these are not located within the gene itself.
